# Integrative Comparison of Burrows-Wheeler Transform-Based Mapping Algorithm with de Bruijn Graph for Identification of Lung/Liver Cancer-Specific Gene

**DOI:** 10.4014/jmb.2110.10017

**Published:** 2021-12-25

**Authors:** Atul Ajaykumar, Jung Jin Yang

**Affiliations:** 1 Department of Information, Communication and Electronics Engineering, The Catholic University of Korea, Bucheon 14662, Republic of Korea; 2 Department of Computer Science Engineering, The Catholic University of Korea, Bucheon 14662, Republic of Korea

**Keywords:** mRNA sequencing data, lung/liver cancer, cancer-specific biomarker, bowtie2, Kallisto, mapping comparison

## Abstract

Cancers of the lung and liver are the top 10 leading causes of cancer death worldwide. Thus, it is essential to identify the genes specifically expressed in these two cancer types to develop new therapeutics. Although many messenger RNA (mRNA) sequencing data related to these cancer cells are available due to the advancement of next-generation sequencing (NGS) technologies, optimized data processing methods need to be developed to identify the novel cancer-specific genes. Here, we conducted an analytical comparison between Bowtie2, a Burrows-Wheeler transform-based alignment tool, and Kallisto, which adopts pseudo alignment based on a transcriptome de Bruijn graph using mRNA sequencing data on normal cells and lung/liver cancer tissues. Before using cancer data, simulated mRNA sequencing reads were generated, and the high Transcripts Per Million (TPM) values were compared. mRNA sequencing reads data on lung/liver cancer cells were also extracted and quantified. While Kallisto could directly give the output in TPM values, Bowtie2 provided the counts. Thus, TPM values were calculated by processing the Sequence Alignment Map (SAM) file in R using package Rsubread and subsequently in python. The analysis of the simulated sequencing data revealed that Kallisto could detect more transcripts and had a higher overlap over Bowtie2. The evaluation of these two data processing methods using the known lung cancer biomarkers concludes that in standard settings without any dedicated quality control, Kallisto is more effective at producing faster and more accurate results than Bowtie2. Such conclusions were also drawn and confirmed with the known biomarkers specific to liver cancer.

## Introduction

Lung and liver cancers are included in the top 10 leading causes of cancer death in both men and women worldwide. Lung cancer is the first-leading cause of cancer death, while liver cancer is the most rapidly increasing cancer in the United States [1, 2]. Although the death rate for lung cancer has decreased over the years, the survival rate for regional and distant cancer stages is only 28 and 4%, respectively. Since early detection and therapeutic monoclonal antibody therapy-specific cancer surface protein have been shown to reduce the mortality of both lung and liver cancer, identification of the genes encoding the surface proteins explicitly expressed in lung or liver cancer tissue is critical for the development of new diagnosis strategies as well as novel therapeutics [3-9].

The decrease in sequencing cost and the advancement of new mRNA sequencing technology have led to an increase in mRNA sequencing data about various cancer tissues in the domains of genetics and genomics. Multiple algorithms and tools can be used efficiently to align and quantify these sequencing data to genomic features such as genes and transcripts. Initially, the algorithms used to map sequence reads to reference transcriptome were based on hash tables [10], then a rise in aligner tools whose algorithm is based on the Burrows-Wheeler transform (BWT) [11] has been observed. This rise was due to BWT being computationally efficient and combined with a Full-text Minute (FM) index causing reduced memory usage. Based on our reference [12, 13], which compares different BWT tools (Bowtie2 [14], HISAT2 [15], BWA [16]), we selected Bowtie2 since it was the most robust and used the least memory in the real dataset (HISAT2 used the least memory in the simulated dataset). STAR [17] is another popular alignment tool but is infamous for memory consumption [18]. In our manuscript, we also consider runtime and memory utilization, which could affect hardware requirements. Both Bowtie2 and Kallisto are known to have a small memory bandwidth. The selection of aligner tools is essential for the final analysis of the new biomarker specific to lung/liver cancer tissue. The output of the alignment could be dependent on the read length, read quality, and other factors. Depending on the scale of the project, it may not be efficient to add quality control measures at multiple levels. The new tool, like Kallisto, uses pseudo alignment exercising the de Bruijn graph and becomes popular due to their shallow alignment time and low memory usage.

Our initial analysis is based on simulated data to show which tool could align with high TPM values to unique transcripts. For the actual experiment data, we used mRNA sequencing data about lung cancer tissue, the evaluation of these two tools, one based on BWT (Bowtie2) and the other on the de Bruijn graph [19] (Kallisto [20]). We attempted a comparison of their transcript TPM values. The evaluation was attributed to the fact that no prior custom quality control was added.

Taking our results together, a quick alignment favors the output of Kallisto since it has inbuilt quality control, and the Kallisto tool is better for getting faster results more accurately than Bowtie2. In the case of simulated data, Kallisto was able to detect unique transcripts with high TPM values. These results were confirmed using mRNA sequencing data about liver cancer cells and the known liver cancer-specific biomarkers.

## Materials and Methods

### Dataset

The simulated mRNA sequencing data were generated using the DWGSIM tool. The sequencing data generated was 1 million base pairs in length, taking 100 base pairs at a time in a paired end-setting. The simulated data were based on the Illumina device using the tool settings to get similar output to the cancer data used later. The output fastq files were then processed with Kallisto or Bowtie2 to get TPM values. In the case of Kallisto, the TPM values were directly generated. For Bowtie2, the read counts were prepared using the featureCounts function available in Rsubread [21] package in R, and the output of feature counts is saved with the transcript length. Both of these values were then used to compute the TPM value as shown in the Methods section.

For actual cancer data, mRNA sequencing data for lung and liver cancer tissue and the corresponding normal tissue were downloaded from Sequence Read Archive (SRA) [22]. mRNA was sequenced using Illumina Hiseq 2000 in a paired end-setting at 100bp. The mRNA sequencing raw data were prepared using parallel-fastq-dump with a split file parameter in paired-end reads. The fastq files were processed in the same process as with the simulated data.

### Bowtie2 (Burrows-Wheeler Transform)

Initially, the sequence alignment was done based on hash tables. Hash tables have high computation power and long run times, which has led to an increase in the use of more efficient algorithms like BWT. The BWT algorithm creates a suffix array with the transcript, which is lexicographically sorted. BWT stores the last column as the prefix tree of the genome.

Bowtie2 utilizes a BWT backtracking strategy to perform a depth-first search through the suffix trie that holds all suffixes of the reference transcriptome. It matches the first alignment that satisfies specific criteria found. Bowtie2 tool was used to process the downloaded sequence fastq file. The Bowtie2 index was built using the human hg38 reference transcriptome from ensemble reference transcriptomes (https://uswest.ensembl.org/info/data/ftp/index.html) using default settings. The raw fastq files were processed using the Bowtie2 tool with the parameter to align for paired-end reads. The SAM file with a hg38 genomic annotation file was available from (https://github.com/pachterlab/kallisto-transcriptome -indices/releases) homo sapiens annotation folder, and feature counts function available in Rsubread package in R. To get the required output as transcripts, feature counts require additional parameters. The additional parameters used for feature counts were, GTF AnnotationFile set as TRUE, isPairedEnd set as TRUE, GTF attrType set as "transcript id". The transcript level read counts were generated, which was further processed to get transcript level TPM values in python. Additionally, this process was tried in a local mode setting where soft clipping to the reads before aligning is done.

### Kallisto (de Bruijn Graph)

Aligner tools are used to align the sequence reads to a reference genome or transcriptome database. Kallisto is an aligner that uses de Bruijn graphs to construct its index and makes a pseudo alignment on this graph to quickly align the sequence to the transcriptome during the alignment phase.

**Fig. 1:**
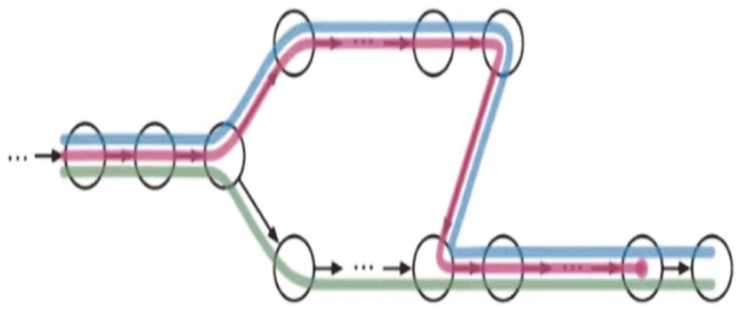
De Bruijn graph is a directed graph with overlapping nodes, where the nodes are k-mers. As an example, here we have 3 different transcripts, shown by color.

**Fig. 2:**
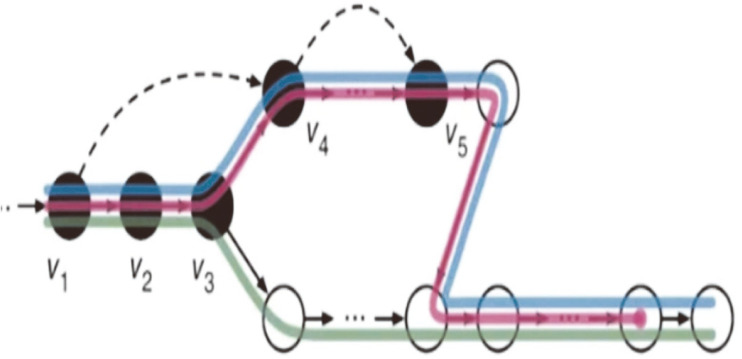
Pseudoalignment is when the matching skips similar k-compatibility classes. Since the k-compatibility class value is the same, those transcripts share similar sequences. So, the algorithm skips the similar nodes for efficiency.

For alignment, the tool requires an index that associates the sequence reads to the gene/transcript. In the case of Kallisto, the de Bruijn graph was used to build the index. Each node in the graph represents k-mers, the sequences of base pairs, as seen in Fig. 1. During the alignment to the transcriptome, Kallisto does a pseudo-alignment; if connected nodes have the same k-compatibility class, it skips those until the value of the k-compatibility class changes, as seen in Fig. 2. By default, Kallisto makes the index with a k-mer size of 31. If the base pair read length is lower than 31, the index must be built with the appropriate k-mer size.

Kallisto provides the prebuilt indexes based on the hg38 human reference transcriptome. Kallisto quant was used to quantify the abundance of transcripts. The index file, output folder, and raw file need to be built to be processed. In the case of paired-end reads, both raw files should be given. Three files in the output folder were prepared, but mainly the abundance.tsv file, which holds the transcripts TPM values, was examined. Kallisto was used to process all the fastq files with an index file. The prebuilt index was downloaded from the Kallisto manual (https://pachterlab.github.io/kallisto/manual) and built on the same ensemble reference transcriptome used by Bowtie2. This index is created on a 31 k-mer length, which is the default. Additional parameters of paired-end reads were added, and both paired files were processed together. Kallisto directly gave the transcript TPM values from raw fastq files. The output abundance.tsv was taken into python for further analysis and comparison with Bowtie2’s output.

### Cosine Similarity

**Fig. 3:**
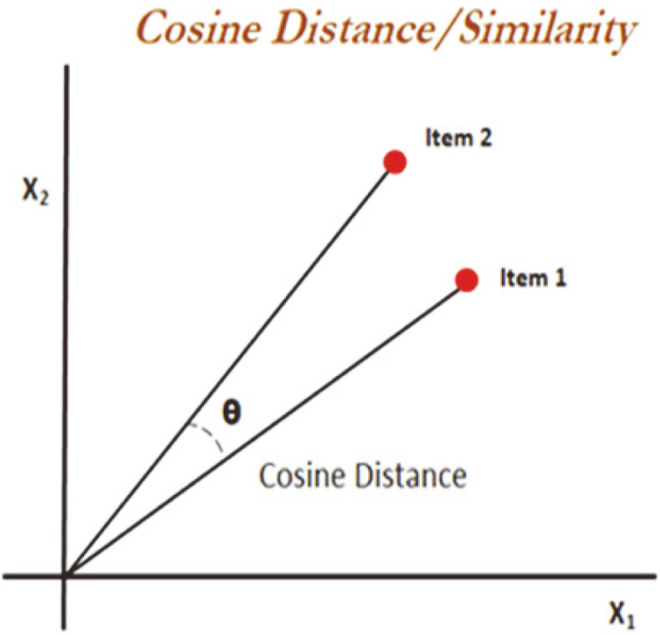
Cosine similarity between 2 items is the θ angle between the items.

Cosine similarity measures the similarity between two vectors by calculating the angle θ between them (Fig. 3). Given two vectors A and B, the similarity can be calculated by their dot product and magnitude as given with Eq. (1):

$$\cos(\theta )=\frac{A\cdot B}{\left|A||B\right|}=\frac{\sum_{i=1}^{n}A_{i}B_{i}}{\sqrt{\sum_{i=1}^{n}A_{i}^{2}}\sqrt{\sum_{i=1}^{n}B_{i}^{2}}}$$ (1)

A value of 1 signifies complete similarity, while that of –1 shows they are entirely dissimilar. Cosine similarity between the standard transcript TPM values was used. For Kallisto, the tool directly outputs in transcript-TPM values, and in the case of Bowtie2, the output was processed through R and python.

### Transcripts Per Kilobase Million

The TPM value was used to normalize the reads counts by normalizing for gene length first and then normalizing for sequencing depth.

To calculate TPM:

1. Divide the length of each transcript into kilobases. This gives us the reads per kilobase (RPK)

2. Sum up all the RPM values and divide this by 1,000,000. This gives us the “per million” scaling factor.

3. Divide the RPK values by the per million scaling factors to get TPM values for the transcript.

### DWGSIM

DWGSIM is a whole genome simulator for next-generation sequencing technologies. The tool DWGSIM was used to generate the simulated sequencing data. The tool can be downloaded from the Linux terminal using ‘apt-get dwgsim’ or downloaded from GitHub (https://github.com/nh13/DWGSIM).

### Filtering the Genes Encoding the Plasma Membrane Protein

The location of the proteins encoded by the candidate genes inside the cells was analyzed by Gene Cards. The information about gene ID, functional description, location in the genome, and intracellular localization are provided by Gene Cards (https://www.genecards.org/). Eleven intracellular location codes such as plasma membrane, extracellular space, lysosome, cytoskeleton, endosome, Golgi apparatus, mitochondrion, peroxisome, endoplasmic reticulum, cytosol, nucleus, and 0-5 possibility score are assigned to the gene. The genes with a score of 4 or 5 of plasma membrane value were selected to filter out the gene encoding the plasma membrane protein.

### Validation Process

To validate our claims, the TPM values in a comparative way (highly expressed transcripts in one tool and not the other) were individually examined to validate which tool is better equipped to directly give close to accurate results. For example, if a transcript is highly expressed in the given tumor tissue, it was evaluated as to which tool was more accurate in aligning high fold change for that transcript. In lung cancer tissue, the candidate transcripts were analyzed to match known biomarkers such as CA9, GPR87, CA12, etc.

Further confirmation of the above process was made with mRNA sequencing data about liver cancer tissue and the known biomarkers specific to liver cancer, such as PROM1, CD44, ITGA6, etc. Additionally, the analysis was also performed to check any relation between the subcellular localization and the alignment of biomarkers. The localization was taken from uniport and cross-validated with genecards.org. Some of the known biomarkers have localization scores for the plasma membrane as 4. These biomarkers were also added to the evaluation list.

### Data Availability

Raw sequencing data are publicly available through the GEO database (GSE70089).

## Results

**Fig. 4:**
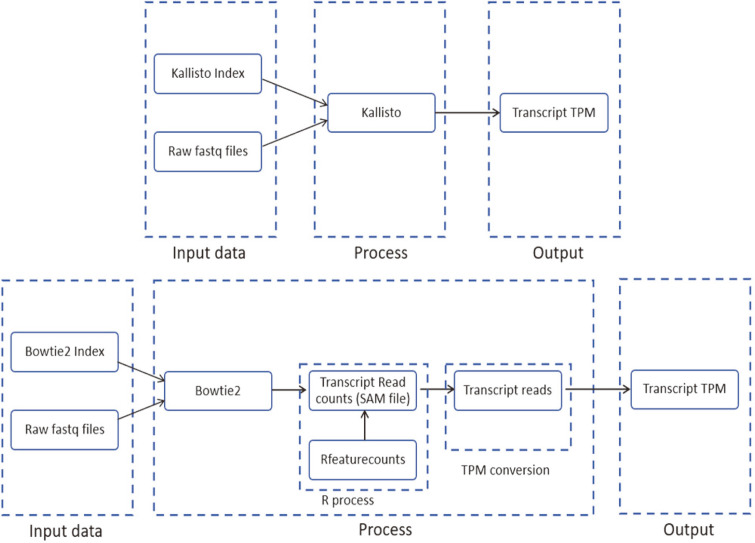
Process architecture for Kallisto and Bowtie2 for the alignment of transcripts and calculation of TPM values. The pipeline on top shows the input, process, and output for Kallisto. Since no additional steps are being carried out by us, the pipeline is direct. In case of Bowtie2, the tool does not directly give the output as transcript TPM values, so we added an additional R process to handle the transcript read counts and TPM conversion to convert the expression levels to TPM.

For our overall workflow, each tool has its pipeline. Our objective was to compare the final expression values derived from both tools using the duplicate input files. Both tools are run on the same system to make the comparisons fair. The simulated and provided input files are in Fastq format. Fastq format is a text format for biological sequences and their quality score. Both tools use the same input raw files but have separate pipelines, as shown in Fig. 4. Separate pipelines are required since the output of both tools is different, but to make our comparison, we needed to process both the tool outputs to the required transcript TPM data. The same human reference transcriptome was used to build the indexes for both tools to maintain consistency for comparison. Bowtie2 has additional steps to get the TPM value as the alignment produces a SAM output. The SAM file received from Bowtie2 was processed in R to get the read counts, further processed in python to get the TPM values. Kallisto directly gives the output in transcript TPM values. Once the final TPM values were obtained from both pipelines, the analysis was done in python. The TPM values are analyzed based on their alignment rate, the number of transcripts aligned, and in the case of cancer cells, we check how many known cancer biomarkers the tools could align.

### Generation of TPM Values from Simulated Sequencing Data

Before we analyzed the actual cancer data, we ran both tools on simulated sequencing data to analyze their outputs. The tool DWGSIM was used to generate raw sequencing reads in a paired- end setting. The option for generating simulated reads from Illumina was selected to closely resemble the reads generated from the cancer data since they were processed on an Illumina device. A genome sequence of 1 million nucleotides with 100bp reads and 10 million nucleotides with 100 bp were generated. The output of this process yielded two fastq files since it was in a paired-end setting. Genome sequences of different lengths were generated to check whether the results were consistent with longer sequences.

The fastq files were initially processed with Bowtie2. The output of Bowtie2 for the simulated data gave a SAM file which was processed in R to get the expression levels. Since we compare the TPM values, the expression levels are converted to TPM values in python.

For the genome sequence with a length of 1 million reads, the two fastq files generated are processed using the tool Kallisto. Since Kallisto directly gives output in TPM, no further processing is required. Bowtie2 was able to align to 41388 transcripts while Kallisto aligned 110762 transcripts. There were 39972 transcripts present in both outputs. Total unique transcripts aligned were 112128. The cosine similarity between TPM values of common transcripts was 79.2%.

Since our interest lies in discovering genes or transcripts with high expression changes, we look at the top expressed transcripts taken from both tools and compare them to one another. The top 1000 transcripts ordered by TPM values in descending order of Bowtie2 were accepted. We observed that all 1000 transcripts were also aligned with Kallisto. The cosine similarity between them was 98.9%. Next, the top 1000 transcripts in descending order of TPM values for Kallisto were taken. Here, we observed that only 12 from these 1000 transcripts were aligned with the Bowtie2 tool. Based on this analysis, it was observed that Kallisto also aligned transcripts with high TPM values from Bowtie2, but many transcripts having high TPM values from Kallisto were not aligned by Bowtie2.

To check for consistency, we simulated sequencing data with higher read counts. The same process was followed for the genome sequence of 10 million reads. Bowtie2 aligned to 41388 transcripts, while Kallisto aligned to 134811 transcripts. Common transcripts from both tools were 40501. There were 135648 unique combined transcripts when taking individual transcripts from both tools. The cosine similarity between the TPM values of both tools was 76.28%.

Like the genome analysis with 1 million reads, the top 1000 TPM values ordered by both the tools were compared for 10 million reads. We observe that the top 1000 transcripts from Bowtie2 based on TPM value were present in Kallisto, but the top 1000 from Kallisto were not present in Bowtie2. The counts of aligned transcripts are given in Table 1.

This observation shows Kallisto has better transcript alignment since it could align to more unique transcripts with high TPM values and is more helpful in discovering novel unique biomarkers.

### Generation of TPM Values for Lung Cancer Tissue mRNA Using Bowtie2

Evaluation of both tools on actual data is required since simulated data do not accurately represent the significance of finding highly expressed genes. For this, we look at lung cancer sequencing data. To get TPM values for the transcripts, the sequence data need to be aligned to the transcripts. Bowtie2 was used to make the alignment. The sequence read files were downloaded from SRA using the fastq-dump tool recommended on the NCBI website. We got two separate fastq files for every sample as the reads were processed on the Illumina platform with a paired-end reads layout. The fastq file was processed with Bowtie2. The output of this process yielded a SAM file with an approximate 85% alignment rate. On average, it took Bowtie2 125 minutes to make the alignments. The SAM file was processed in R to get the expression levels using the function featureCounts available in the package Rsubread. Additional parameters were used with feature counts to add the hg38 annotation file and attribute type as transcript id to give output in terms of transcripts (the default is genes). The final output was written to a CSV file, which was further processed in python. The TPM value calculation is mentioned in the Methods section. TPM values were also calculated by running Bowtie2 in local mode, which clips the reads. A similarity score of 0.9993 was obtained between clipped and non-clipped reads. In further analysis, the non-clipped reads data were used without the local parameter to adhere to default parameters to compare both tools.

### Alignment of Lung Cancer Tissue mRNA Using Kallisto

The lung cancer sequencing data were also processed using Kallisto. Kallisto takes in the fastq files and directly gives the output in transcript TPM values. To analyze the cancer cell data, which is in a nucleotide sequence format, it is necessary to align them to the human reference transcriptome. On average, the Kallisto process aligned the transcripts and calculated their TPM values in 6 min and with an approximately 87.3% alignment rate. Based on performance, Kallisto aligned to more transcripts and took significantly less time to run. We also observed that Kallisto was able to align with more transcripts.

### Filtering the Surface Protein-Encoding mRNA from the Lung Cancer Cells

To evaluate which tool is better suited for finding marker genes, we cross-checked the results with known surface markers and how well the tools were able to detect them from the cancer data. The plasma membrane proteins present on the surface of lung cancer cells are valuable targets for the development of monoclonal antibodies (mAb), which are known to be the most effective reagent for clinical application and basic mechanism study. The technologies and protocols for the generation and large-scale production of mAb have been well established. The database to localize the protein encoded by the gene in the cells was downloaded from genecards.org. Based on the intracellular localization of the protein encoded by the gene, the genes with high value for plasma membrane were filtered. The genes such as CA9, CA12, KIT, THY1, and others, as shown in Table 2, which was reported to encode the lung cancer cell-specific surface protein, were included in the biomarker genes, implying that our strategy to identify the novel gene specifically expressed in lung cancer cells was legitimate.

### Comparison of the Transcripts Aligned by Both Tools

Next, we looked at analyzing the TPM values obtained from both tools. This gives an insight as to which tool provides a better outcome in terms of transcripts. In all cases, Kallisto was able to align to more distinct transcripts with very low TPM values. This could be an advantage depending on the case of the project for which the alignment was made. (But in our case, since the fold change was used, Kallisto fold-change values became exponentially higher than that of Bowtie2). TPM values from both outputs were taken and compared in python, revealing that 34897 transcripts were common in both outcomes. The cosine similarity between both the outputs lay at 0.896 in lung cancer cells and 0.672 for normal lung cells. The cosine similarity showed that for both tools, the transcripts were similarly expressed.

In the case of analyzing multiple samples, comparison was made by taking common transcripts using inner joins or taking all transcripts by using the outer join of all processed files. Many transcripts aligned from tumor and non-tumor cells were compared. The counts of transcripts for tumor or non-tumor cells among the tools were compared by taking common transcripts among the multiple cell samples, as shown in Table 3. There was an overlap of 86.4% of transcripts between the tumor and non-tumor data for Kallisto and 86.3% overlap for Bowtie2. Also, 43.6% of transcripts (common from both tumor and non-tumor) of Kallisto were present in Bowtie2 transcripts.

The counts for all transcripts were also compared, which included the unique transcripts aligned in the individual cell samples, as shown in Table 4. The common transcripts were calculated by taking inner joins between tumor and non-tumor, while unique transcript count was calculated using a full outer join. The data were split based on the tool used and cell type. By taking all aligned transcripts and comparing them to common transcripts among the cells, many transcripts were observed to be uniquely aligned for the individual cells. The data were ordered by TPM value in descending order for both tools and plotted to a graph.

**Fig. 5:**
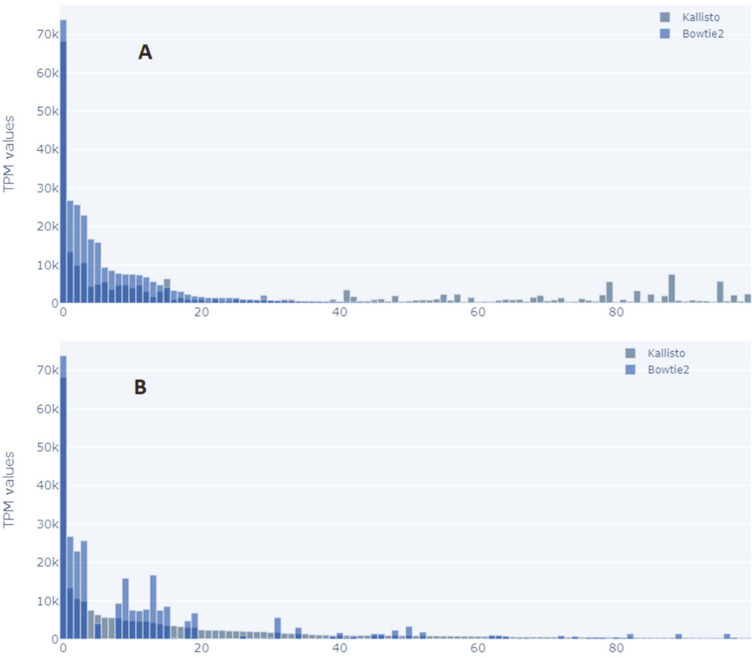
Comparison of top 100 TPM values of tumor cells taken in ascending order by the tools to show if Bowtie2 can align the top 100 from Kallisto and vice versa.

**Fig. 6:**
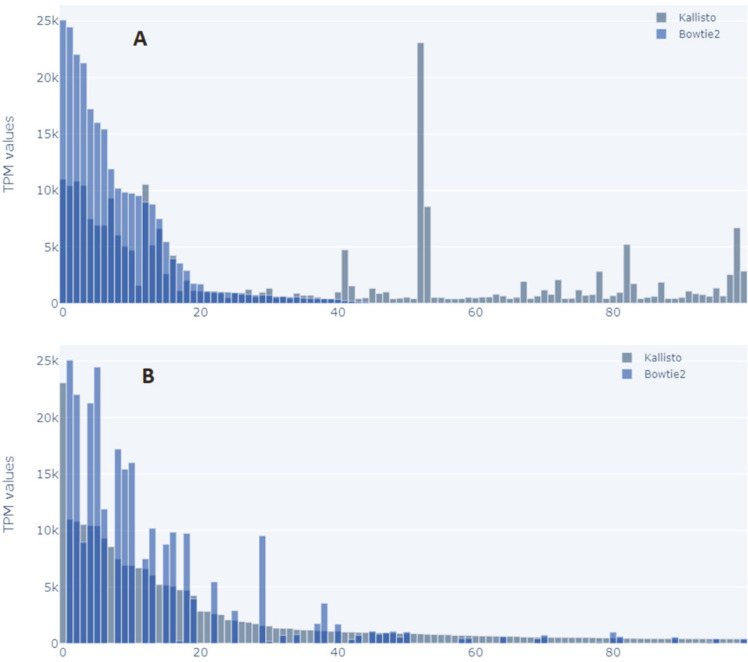
Comparison of top 100 TPM values of non-tumor cells taken in ascending order by the tools to show if Bowtie2 can align the top 100 from Kallisto and vice versa.

We observed that Kallisto could align a lot more transcripts in all cases. Most of these different transcripts had a lower value. As shown in Figs. 5 and 6, TPM values were ordered in descending values for both tools in the case of tumor cells and non-tumor cells. Based on this, it was noted that some highly expressed transcripts in Kallisto were not represented in transcripts processed by Bowtie2.

### Integrative Analysis of Known Lung Cancer Biomarkers Using Both Tools

To analyze which tool better aligns the genes to the known set of biomarkers for lung cancer, further analysis was done on the processed files, and the fold change of the transcript TPM values was compared. First, the transcripts associated with the biomarker genes were filtered. The number of filtered transcripts is shown in Table 5. Since there were multiple tumor and non-tumor cell samples, the average value of lung cancer cell and non-cancer cell TPM output was taken, and their fold change was calculated accordingly. The assessment was made by considering FC > 2 as being highly expressed. If any single transcript for the biomarker gene satisfied this condition, it was noted that the gene is highly expressed. In case the gene is highly expressed using the tool, it was denoted with ‘Y.’ If the conditions were not matched, it was marked with ‘N.’ In case there were no transcripts aligned for the biomarker, it was denoted by ‘ –’. Biomarkers were assessed with these conditions, as shown in Table 6.

For most of the biomarkers, both tools gave high TPM values. In some cases where Bowtie2 could not align any transcripts associated with the biomarker gene, Kallisto could align those transcripts. Based on the criteria to show which tool could align the biomarker correctly, Kallisto got 11 biomarkers while Bowtie2 got nine biomarkers. The subcellular localization of the proteins encoded by the genes was also considered. We found that high fold change values were obtained for both tools. As observed in this study, Kallisto could align more transcripts for the biomarkers specific to lung cancer. For most of the biomarkers, Kallisto aligned multiple transcripts to the gene. For example, in the gene TMPRSS4, Bowtie2 aligned it to 1 transcript while Kallisto aligned it to 4 transcripts.

### Confirmation of Our Results by Using the Known Biomarkers Specific to Liver Cancer

To confirm the results of our study using the data about lung cancer and lung cancer-specific surface biomarkers, additional mRNA sequencing data related to liver cancer cells and normal liver cells were processed. The raw fastq files were downloaded from SRA (accession numbers provided in data availability statement). The fastq-dump tool was used to download the data from SRA with a split function, which divides the data into two files sequenced in a paired-end layout. Three samples of liver cancer cells and three samples of normal liver cells were downloaded for analysis for liver cancer data. Bowtie2 gave an average alignment rate of 85.95% for liver cancer cells and 83.15% for normal liver cells. The SAM file was further processed in R to get the read counts with function feature counts available in the Rsubread package. Additional parameters to use external hg38 annotation file and output as transcripts were added. Kallisto gave an average alignment rate of 86.5% for liver cancer cells and 91.2% for normal liver cells.

The counts were compared for the liver cancer data. When taking inner join with both tumor and non-tumor for Kallisto, 82.6% of transcripts were common while Bowtie2 has a 79% overlap. The count comparison of liver cancer data is shown in Table 7 (only common transcripts from all cells) and Table 8 (unique transcripts among all cells).

It was observed that Kallisto aligns with more transcripts for liver cancer, as seen with lung cancer data. The known biomarkers specific to liver cancer cells are shown in Table 7. Table 8 and Table 9 showed the number of transcripts aligned by both tools taking unique transcripts across the samples and total transcripts across the samples, respectively. Consistent with the lung cancer case results, as shown in Table 10, Kallisto aligned more transcripts for liver cancer data as well. The biomarker gene SALL4, which is localized in the nucleus, had a low fold change value. Table 11 shows a comparison of the tools in finding the biomarkers based on high fold change. Transcripts aligned with Kallisto gave six biomarkers, while Bowtie2 produced only two biomarkers. Also, Bowtie2 was unable to align any transcript to the biomarker CD24.

Consistent with the results from mRNA sequencing data about lung cancer cells and their specific genes encoding the surface proteins, Kallisto was demonstrated to perform better than Bowtie2 in aligning transcripts related to the liver biomarker genes and aligns more transcripts for the biomarker genes than Bowtie2.

## Discussion

Alignment tools are essential in biomedical strategy to quantify mRNA sequence data and identify the novel genes specifically expressed in the target cells. This study compared two different alignment tools that use different underlying algorithms for aligning biological sequence data. Bowtie2 is an alignment tool based on the Burrows-Wheeler transform, while Kallisto makes its alignment based on the de Bruijn graph algorithm.

The initial comparison was made by observing both tools' number alignments and TPM values on the simulated data of 1 million and 10 million reads. Two different read lengths were taken to check for consistency based on different read lengths. The simulated data settings were kept to closely resemble the raw sequencing reads generated for the actual cancer data. This was done to make sure similar results could be drawn from both types of data. Kallisto aligned approximately ~3 times more transcripts than Bowtie2 in the case of the simulated data. We also observed that Kallisto aligned more unique transcripts with high TPM values than Bowtie2.

For the actual cancer data, our observation was based on the number of transcripts and the time taken for the tools to make the alignments. Kallisto, on average aligned up to 60% more transcripts than Bowtie2. Kallisto made these alignments in ~6 min while Bowtie2 took an average of 125 min. In this regard, Kallisto aligned more transcripts in all cases and did it in significantly less time than Bowtie2. The next part of the evaluation was done by examining which alignment tool could align better with the known biomarkers specific to two different cancer types (lung and liver cancer). A set of tumor and non-tumor tissues from lung cancer patients was used to determine which tool could select the transcripts expressed more in the cancer tissue cells by fold change. From this, it was observed that Kallisto had a higher fold change value for most of the biomarker transcripts. This result was further validated using tumor and non-tumor liver cancer tissues, where Kallisto again produced better results. It was also noted that there were some biomarkers to which Bowtie2 failed to align any transcript, but Kallisto could.

There are many technical features during sequence reads that may complicate running and getting accurate results directly with the aligner tools. Out of the box, Kallisto is seen to get better results with minimum interference from the user. In this case, the interference includes a separate tool/script for quality control of the biomedical sequence data. This difference in TPM/read counts between Kallisto and Bowtie2 could be accounted for by Kallisto having bias correction inbuilt, which acts as quality control. Based on comparing the known biomarkers and those biomarker TPM values obtained from both tools, it can be concluded that without any dedicated quality control and on standard settings Kallisto tool is better for getting faster results more accurately. Therefore, the usage of the Kallisto algorithm is essential to identify the novel genes specifically expressed in the target cells, which should be the fundamental core asset in developing diagnostic and therapeutic reagents for the patients.

## Figures and Tables

**Table 1:** Comparison of transcripts aligned by both tools taking the same simulated sequencing data of 1M and 10M nucleotide lengths.^a^

Tool	Transcripts aligned (1M reads)	Transcripts aligned (10M reads)
Kallisto	110762	134811
Bowtie2	41388	41388
Unique	112128	135648

^a^Unique values are taken by combining transcripts aligned by both tools and removing duplicate transcripts.

**Table 2:** Biomarker genes specific to lung cancer cells.

Biomarker	Ensembl gene ID	Transcript count	Localization	Localization score
CA9	ENSG00000107159	4	Plasma membrane, nucleus	5,4
CA12	ENSG00000074410	6	Plasma membrane	5
CT83	ENSG00000204019	1	Plasma membrane	4
DSG3	ENSG00000134757	1	Plasma membrane	5
FAT2	ENSG00000086570	2	Plasma membrane, golgi	4,3
GPR87	ENSG00000138271	2	Plasma membrane	4
KISS1R	ENSG00000116014	3	Plasma membrane	5
LYPD3	ENSG00000124466	4	Plasma membrane, extracellular	5,5
SLC7A11	ENSG00000151012	2	Plasma membrane	5
TMPRSS4	ENSG00000137648	20	Plasma membrane	4
PROM1	ENSG00000007062	20	Plasma membrane, extracellular	5,5
PLAUR	ENSG00000011422	16	Plasma membrane, extracellular	5,5
KIT	ENSG00000157404	4	Plasma membrane	5
ABCG2	ENSG00000118777	5	Plasma membrane, nucleus	5,5
THY1	ENSG00000154096	10	Plasma membrane	5

**Table 3:** Comparison of the number of transcripts that were aligned for both tools by taking common transcripts of lung cancer.^a^

Tool	Tumor cell	Non-tumor cell	Common
Kallisto	91940	92420	79924
Bowtie2	54743	55485	47924
Common	41041	41301	34897

^a^Common transcripts are transcripts aligned in both sets.

**Table 4:** Comparison of the number of transcripts for both tools by taking all transcripts that were aligned of lung cancer.^a^

Tool	Tumor cell	Non-tumor cell	Unique
Kallisto	141830	139623	153435
Bowtie2	90468	88317	100156
Unique	163291	160578	176859

^a^Unique values are taken by combining transcripts aligned by both tools and removing duplicate transcripts.

**Table 5:** Lung cancer biomarker transcripts aligned count using both tools.

Tool	Transcripts aligned	Transcripts filtered
Kallisto	126	66
Bowtie2	57	23
Common^a^	57	22

^a^Transcripts aligned by both tools.

**Table 6:** Evaluation of both tools on how correctly they can align to lung biomarker genes.

Biomarker	Bowtie2	Kallisto
CA9	-	Y
CA12	Y	Y
CT83	Y	Y
DSG3	N	N
FAT2	N	N
GPR87	Y	Y
KISS1R	Y	Y
LYPD3	Y	Y
SLC7A11	Y	Y
TMPRSS4	Y	Y
PROM1	-	Y
PLAUR	N	N
KIT	Y	Y
ABCG2	N	N
THY1	Y	Y

**Table 7:** Biomarker genes specific for liver cancer.

Biomarker	Ensembl gene ID	Transcript Count	Localization	Localization score
PROM1	ENSG00000007062	20	Plasma membrane, extracellular	5,5
CD44	ENSG00000026508	39	Plasma membrane	5
ITGA6	ENSG00000091409	2	Plasma membrane	5
THY1	ENSG00000154096	10	Plasma membrane	5
ANPEP	ENSG00000166825	9	Plasma membrane	5
EPCAM	ENSG00000119888	6	Plasma membrane, extracellular	5,4
ABCG2	ENSG00000118777	5	Plasma membrane, nucleus	5,5
CD24	ENSG00000272398	8	Plasma membrane	5
SALL4	ENSG00000101115	5	Nucleus	5
ICAM1	ENSG00000090339	5	Plasma membrane	5

**Table 8:** Comparison of the number of transcripts that were aligned for both tools by taking common transcripts of liver cancer.^a^

Tool	Tumor cell	Non-tumor cell	Common
Kallisto	83351	79634	68901
Bowtie2	49201	45114	39131
Common	37055	33573	29127

^a^Common transcripts are transcripts aligned in both sets.

**Table 9:** Comparison of the number of transcripts for both tools by taking all transcripts that were aligned with liver cancer.^a^

Tool	Tumor cell	Non-tumor cell	Common
Kallisto	137284	132227	148301
Bowtie2	86160	79131	93328
Common	157678	150915	170002

^a^Unique values are taken by combining transcripts aligned by both tools and removing duplicate transcripts.

**Table 10:** Biomarker transcripts aligned with both tools for liver cancer tumor and non-tumor cells together.^a^

Tool	Transcripts aligned	Transcripts filtered
Kallisto	97	51
Bowtie2	41	16
Common	40	16

^a^Common transcripts are transcripts aligned in both sets.

**Table 11:** Evaluation of both tools on how correctly they can align to liver biomarker genes.

Biomarker	Bowtie2	Kallisto
PROM1	N	N
CD44	N	Y
ITGA6	N	Y
THY1	N	Y
ANPEP	Y	Y
EPCAM	N	N
ABCG2	Y	Y
CD24	-	Y
SALL4	N	N
ICAM1	N	N
